# The Phosphatidylinositol 3,4,5-trisphosphate (PI(3,4,5)P_3_) Binder Rasa3 Regulates Phosphoinositide 3-kinase (PI3K)-dependent Integrin α_IIb_β_3_ Outside-in Signaling*

**DOI:** 10.1074/jbc.M116.746867

**Published:** 2016-11-30

**Authors:** Anthony M. Battram, Tom N. Durrant, Ejaife O. Agbani, Kate J. Heesom, David S. Paul, Raymond Piatt, Alastair W. Poole, Peter J. Cullen, Wolfgang Bergmeier, Samantha F. Moore, Ingeborg Hers

**Affiliations:** From the ‡ School of Physiology, Pharmacology and Neuroscience and; § School of Biochemistry, University of Bristol, Bristol, BS8 1TD, United Kingdom and; the ¶ McAllister Heart Institute and; ‖ Department of Biochemistry and Biophysics, University of North Carolina at Chapel Hill, Chapel Hill, North Carolina 27514

**Keywords:** cell signaling, integrin, phosphatidylinositide 3-kinase (PI3K), platelet, Ras-related protein 1 (Rap1), GAP1IP4BP, Rasa3

## Abstract

The class I PI3K family of lipid kinases plays an important role in integrin α_IIb_β_3_ function, thereby supporting thrombus growth and consolidation. Here, we identify Ras/Rap1GAP Rasa3 (GAP1^IP4BP^) as a major phosphatidylinositol 3,4,5-trisphosphate-binding protein in human platelets and a key regulator of integrin α_IIb_β_3_ outside-in signaling. We demonstrate that cytosolic Rasa3 translocates to the plasma membrane in a PI3K-dependent manner upon activation of human platelets. Expression of wild-type Rasa3 in integrin α_IIb_β_3_-expressing CHO cells blocked Rap1 activity and integrin α_IIb_β_3_-mediated spreading on fibrinogen. In contrast, Rap1GAP-deficient (P489V) and Ras/Rap1GAP-deficient (R371Q) Rasa3 had no effect. We furthermore show that two Rasa3 mutants (H794L and G125V), which are expressed in different mouse models of thrombocytopenia, lack both Ras and Rap1GAP activity and do not affect integrin α_IIb_β_3_-mediated spreading of CHO cells on fibrinogen. Platelets from thrombocytopenic mice expressing GAP-deficient Rasa3 (H794L) show increased spreading on fibrinogen, which in contrast to wild-type platelets is insensitive to PI3K inhibitors. Together, these results support an important role for Rasa3 in PI3K-dependent integrin α_IIb_β_3_-mediated outside-in signaling and cell spreading.

## Introduction

Integrins are a family of heterodimeric cell adhesion receptors that play critical roles in mediating cell adhesion to adjacent cells and to extracellular matrix, thereby contributing to embryonic development, tissue formation, maintenance and repair, immune responses, and hemostasis. These functions are carried out by bidirectional signaling, which allows integrins to finely mediate cellular responses. Integrins usually exist in a low affinity state but upon cellular stimulation will enter a high affinity ligand-binding state through a process called inside-out signaling. In turn, integrin ligation and clustering triggers outside-in signaling, which is critical in regulating cell spreading and retraction important for cell migration, proliferation, and differentiation.

Platelets provide a highly tractable model for the study of integrins in human tissue, because cell spreading and retraction in platelets is critical for their hemostatic and thrombotic function. Dysregulation of the major platelet integrin α_IIb_β_3_ contributes to the risk/progression of thrombosis in myocardial infarction and ischemic stroke and bleeding in Glanzmann thrombasthenia. In platelets, both inside-out and outside-in signaling from integrin α_IIb_β_3_ leads to the activation of class I PI3K isoforms (1–3), resulting in the generation of the lipid second messenger phosphatidylinositol 3,4,5-trisphosphate (PI(3,4,5)P_3_).^2^ Pharmacological and genetic approaches have revealed that PI3K supports platelet function downstream of multiple receptors to promote platelet aggregation and thrombus stability (4–7). Although details of the PI3K-dependent molecular mechanisms of inside-out signaling in platelets are becoming clearer (8), details of PI3K dependent outside-in signaling, important for cytoskeletal rearrangements to promote cell spreading (4, 9, 10), are more poorly understood. One potential mechanism is for PI3K to enhance activation of the small GTPase Rap1b (4, 11–13), because this has been shown to be critical for normal hemostasis and thrombosis through regulation of both integrin α_IIb_β_3_ inside-out and outside-in signaling (14–17).

Here we addressed the hypothesis that dual Rap and Ras GTPase-activating protein (GAP) Rasa3 (or GAP1^IP4BP^) (8, 18–20) plays a crucial role in PI3K-mediated outside-in signaling from integrin α_IIb_β_3_. We established that: (i) Rasa3 is a major binding partner for PI(3,4,5)P_3_ in human platelets and that its membrane association is up-regulated in a PI3K/PI (3,4,5)P_3_-dependent manner upon platelet activation; (ii) the activity state of Rap1, but not Ras, is regulated by PI3K/Rasa3 in human platelets; (iii) Rasa3 colocalizes with integrin α_IIb_β_3_ in human platelets; (iv) Rasa3 mutants (H794L and G125V), which are expressed in thrombocytopenic mice, lack both Ras and Rap1GAP activity; and (v) that integrin α_IIb_β_3_ outside-in signaling is controlled by Rasa3 Rap1GAP activity and PI3K-mediated inhibition of Rasa3. We therefore propose that integrin α_IIb_β_3_-stimulated PI3K activity contributes to Rap1 activation and cell spreading through inhibition of Rasa3 Rap1GAP activity.

## Results

### 

#### 

##### Rasa3 Is a Highly Abundant Platelet PI(3,4,5)P_3_-binding Protein

**FIGURE 1.**
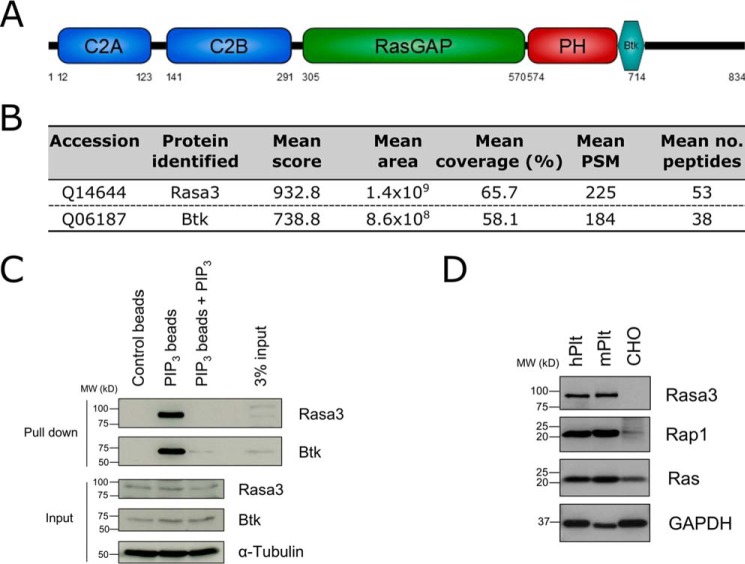
**Rasa3 binds to PI(3,4,5)P_3_ and is highly expressed in platelets.** *A*, Rasa3 domain structure. Rasa3 consists of two N-terminal C2 domains (C2A and C2B), a central RasGAP-related domain (RasGAP), and a C-terminal pleckstrin homology (*PH*)/Btk moiety. *B*, summary of proteomics data. Rasa3 and Btk were captured on PI(3,4,5)P_3_-coated (*PIP_3_*) beads after incubation with human platelet lysate. The total score of the protein is the sum of all peptide Xcorr values for that protein above the specified score threshold. The area is the mean of the area of the three most intense unique peptides matched to that protein. The coverage values indicate the percentages of the protein sequence covered by the identified peptides. *PSM* indicates the total number of identified peptide sequences for the protein, and the *far-right column* shows the number of unique peptide sequences identified for the protein. Rasa3 and Btk were identified in three independent experiments from which the mean values were calculated. *C*, Western blotting confirmation of Rasa3 and Btk captured on PI(3,4,5)P_3_-coated beads. Human platelet lysate was incubated with uncoated control beads, PI(3,4,5)P_3_-coated beads (PIP_3_ beads), or PI(3,4,5)P_3_-coated beads following preincubation of the lysate with competing free PI(3,4,5)P_3_ (PIP_3_ beads + PIP_3_). The input material for each sample was blotted for Rasa3, Btk, and α-tubulin as a loading control. The blots are representative of four independent experiments. *D*, lysates from human platelets (*hPlt*), mouse platelets (*mPlt*), and CHO cells were subjected to immunoblotting to analyze the expression of Rasa3 and its substrates Rap1 and Ras. Input material for each cell type was matched by means of protein assay. The blots are representative of three independent experiments.

One of the mechanisms by which PI3K contributes to platelet function is through the recruitment of PI(3,4,5) P_3_-binding proteins to the plasma membrane. To identify PI(3,4,5)P_3_-binding proteins in human platelets, we used an unbiased affinity proteomics approach utilizing PI(3,4,5)P_3_-coated beads, coupled to LC-MS/MS analysis. This identified the dual Ras/Rap1GAP protein Rasa3 (Fig. 1*A*) as a highly abundant PI(3,4,5)P_3_-binding protein from human platelet lysates (Fig. 1*B*). Indeed, Rasa3 and the well characterized PI(3,4,5)P_3_-specific binding protein Btk were the most abundant proteins identified in our screen.

Western blot analysis confirmed that platelet Rasa3 was captured on PI(3,4,5)P_3_-coated beads with a high abundance and specificity (Fig. 1*C*). Indeed, Rasa3 did not bind to control beads, and preincubation of platelet lysates with competing free PI(3,4,5)P_3_ abolished the capture of Rasa3 on PI(3,4,5)P_3_-coated beads. These approaches established that the binding of Rasa3 to the beads was fully dependent on PI(3,4,5)P_3_ (Fig. 1*C*). Rasa3 is known to be expressed in platelets (21, 22), and we detected Rasa3 and its previously characterized substrates Ras (using a pan-Ras antibody that detects H-Ras, K-Ras, and N-Ras) and Rap1 in both human and mouse platelets (Fig. 1*D*).

##### The Activity State of Rap1, but Not Ras, Is Regulated by PI3K and P2Y_12_ in Platelets

**FIGURE 2.**
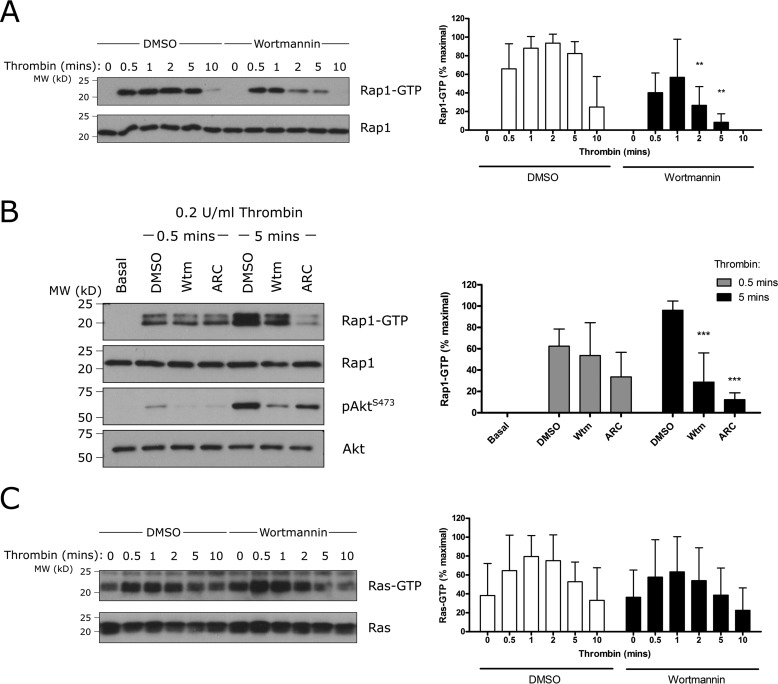
**Sustained Rap1 activation is dependent on PI3K and P2Y_12_, whereas Ras activation is PI3K-independent.** Human washed platelets were incubated with 100 nm wortmannin (*Wtm*), 1 μm AR-C66096 (*ARC*), or vehicle control (0.2% DMSO) for 10 min prior to stimulation with 0.2 unit/ml α-thrombin for the indicated time. Rap1-GTP (*A* and *B*) or Ras-GTP (*C*) was extracted from platelets lysates by GST-RalGDS-RBD or GST-Raf1RBD pulldown, respectively. Pulldown samples were immunoblotted for Rap1 or Ras, and total lysate controls were immunoblotted for Rap1, Ras, or pAkt^S473^ and total Akt. The results are expressed as the percentage of the maximum Rap1-GTP (*A*, *n* = 4; *B*, *n* = 5) or Ras-GTP (*C*, *n* = 6) detected using densitometry. The data are expressed as the means ± standard deviation, and statistical analysis is presented as paired Student's *t* test for each time point to show the effect of wortmannin or AR-C66096 compared with DMSO control (**, *p* ≤ 0.01; ***, *p* ≤ 0.001).

Rap and Ras GTPases are the endogenous targets for Rasa3 (18). Members of both families are highly expressed in platelets and are activated upon stimulation with various agonists (21–23). These data suggest that Rasa3 may regulate platelet function by controlling Rap1/Ras activity levels. The later sustained phase of thrombin-mediated Rap1 activation was strongly reduced in the presence of the pan-PI3K inhibitor wortmannin (Fig. 2*A*). The P2Y_12_ antagonist AR-C66096 also inhibited the later phase of Rap1 activation (Fig. 2*B*), suggesting that sustained Rap1 activation is dependent on autocrine ADP release and subsequent activation of the P2Y_12_/PI3K pathway. The early phase of Rap1 activation was unaffected by wortmannin or AR-C66096, which is in agreement with the role of PLC/CalDAG-GEFI and not P2Y_12_/PI3K in the initial activation of Rap1 (24). In contrast, Ras activation was unaffected by inhibition of PI3K, although there was a trend for maximal Ras activation to be suppressed (Fig. 2*C*). These results demonstrate that sustained Rap1, but not Ras, activation in human platelets is a PI3K-dependent process.

##### Rasa3 Is Predominantly Localized to the Membrane in Close Association with Integrin α_IIb_β_3_

**FIGURE 3.**
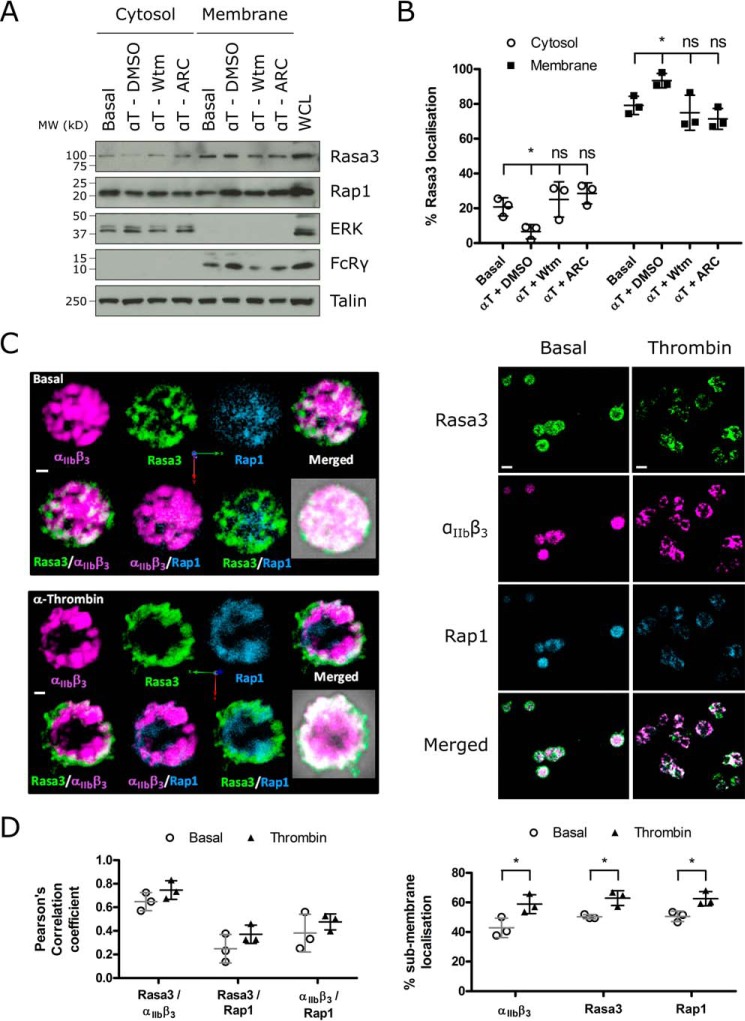
**Rasa3 translocates to the plasma membrane upon agonist stimulation in a PI3K/P2Y_12_-dependent manner and colocalizes with integrin α_IIb_β_3_.** *A*, human washed platelets were incubated with 100 nm wortmannin (*Wtm*), 1 μm AR-C66096 (*ARC*), or vehicle control (0.2% DMSO) for 10 min prior to stimulation with 0.2 unit/ml α-thrombin (α*T*) for 5 min. Cytosolic and membrane fractions were separated by ultracentrifugation and immunoblotted alongside a platelet whole cell lysate (*WCL*) for Rasa3, Rap1, ERK (cytosolic control), FcRγ (membrane control), and talin. The blots are representative of three independent experiments. *B*, quantification of Rasa3 levels in cytosol and membrane fractions from platelet fractionation experiments (*n* = 3). The data are expressed as means ± standard deviation, and statistical analysis shows the effect of thrombin + vehicle control, thrombin + wortmannin, or thrombin + AR-C66096 compared with basal control (*, *p* ≤ 0.05). *C* and *D*, platelets stimulated with 0.2 unit/ml α-thrombin for 5 min or untreated platelets were fixed in 4% paraformaldehyde and spun onto glass coverslips. Adhered platelets were permeabilized and stained with antibodies against integrin α_IIb_β_3_, Rasa3, and Rap1. Localization was identified using Alexa Fluor 568 (integrin α_IIb_β_3_, *magenta*), Alexa Fluor 488 (Rasa3, *green*), and Alexa Fluor 350 (Rap1, *blue*) secondary antibodies. Images were captured using a spinning disk confocal module (PerkinElmer UltraVIEW ERS 6FE confocal microscope) at 100× magnification. *C*, representative extended focus images of three separate experiments. *Scale bars*, 0.5 μm (*left panel*, single cell images) and 2 μm (*right panel*, multiple cell images). *D*, analysis of immunofluorescence data to determine colocalization between Rasa3 and Rap1, Rasa3 and integrin α_IIb_β_3_, and integrin α_IIb_β_3_ and Rap1 (*left panel*), and submembrane localization (*right panel*) was performed using Volocity software, with submembrane defined as 0.5 μm from the outermost point of the cell. The results are expressed as means ± standard deviation (*n* = 3; *, *p* ≤ 0.05).

Previous studies reported that Rasa3 is constitutively membrane-bound by binding phosphatidylinositol 4,5-bisphosphate (PI(4,5)P_2_) (25) but is still sensitive to PI(3,4,5)P_3_ generation in the plasma membrane (26). To determine the effect of thrombin stimulation and PI3K/P2Y_12_ inhibition on the localization of Rasa3 in platelets, we performed fractionation studies. Over 75% of Rasa3 in platelets was found in the membrane fraction of resting platelets (Fig. 3, *A* and *B*). Following thrombin treatment, there was a significant increase in membrane-localized Rasa3, which correlated with a decrease of Rasa3 in the cytosolic fraction (Fig. 3, *A* and *B*). Blocking PI3K activation, with either the generic PI3 kinase inhibitor wortmannin or the PI3K p110β inhibitor TGX-221, or blocking P2Y_12_ with AR-C66096 prevented the thrombin-mediated increase in the membrane localization of Rasa3, with no effect on basal localization (Fig. 3, *A* and *B*, and data not shown). Three-dimensional immunofluorescence studies revealed that Rasa3 and integrin α_IIb_β_3_ show a high degree of colocalization on intracellular vesicles and the plasma membrane, more so than between integrin α_IIb_β_3_ and Rap1 (Fig. 3, *C* and *D*). Rap1 showed a cytosolic distribution with low levels of colocalization with Rasa3 and moved to the membrane region upon thrombin stimulation (Fig. 3, *C* and *D*). Together, these results demonstrate that Rasa3 localization in human platelets is regulated by agonist-stimulated PI3K activity and is closely associated with integrin α_IIb_β_3_.

##### Rasa3 Suppresses Basal and PAR1-mediated Rap1 and Ras Activation in Integrin α_IIb_β_3_-Expressing CHO Cells

**FIGURE 4.**
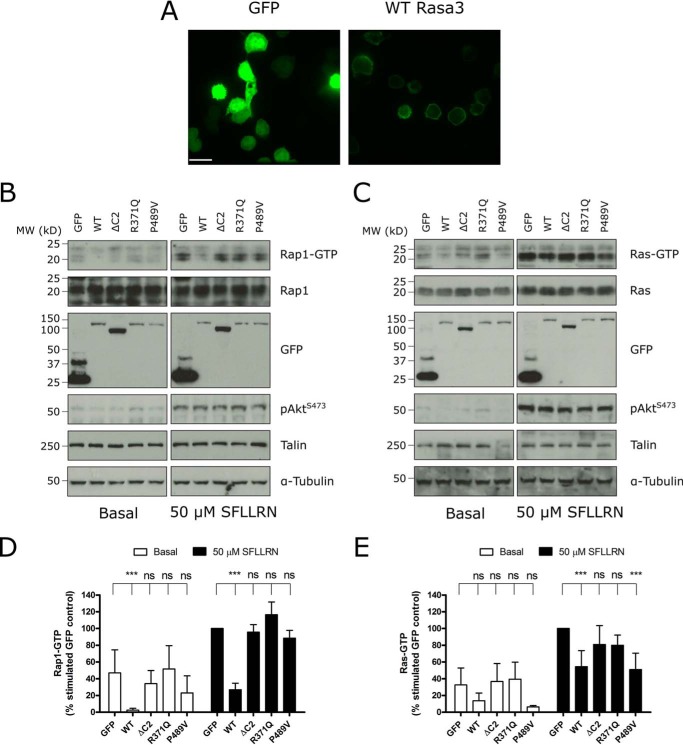
**Rasa3 suppresses Rap1 and Ras activation in integrin α_IIb_β_3_-expressing CHO cells.** *A*, CHO cells that were allowed to adhere to glass-bottomed dishes coated with 0.1 mg/ml poly-l-lysine were transfected with GFP alone (*GFP*) or GFP-conjugated wild-type Rasa3 (*WT Rasa3*). 16 h after transfection, cell medium was replaced with imaging medium, and the cells were imaged on a spinning disk confocal microscope at 63× magnification. The images are representative of three independent experiments. *Scale bar*, 20 μm. *B–E*, CHO cells were transfected with GFP alone or GFP-conjugated WT Rasa3, Rasa3-ΔC2, Rasa3 (R371Q), or Rasa3 (P489V). CHO cells were unstimulated or stimulated with 50 μm SFLLRN for 5 min. Rap1-GTP or Ras-GTP was extracted from platelets lysates by GST-RalGDS-RBD or GST-Raf1RBD pulldown, respectively. Pulldown samples were blotted for Rap1 or Ras, and total lysate controls were immunoblotted for Rap1 or Ras, pAkt^S473^, GFP, talin, and α-tubulin (loading control). *B* and *C*, representative blots from at least four independent experiments. *D* and *E*, quantification of Rap1-GTP (D, *n* = 4–9) or Ras-GTP (E, *n* = 4–7) bands by densitometry, expressed as means ± standard deviation of the percentage of the stimulated GFP control detected. The values are compared with the basal or stimulated GFP control to test for significance (***, *p* ≤ 0.001).

**FIGURE 5.**
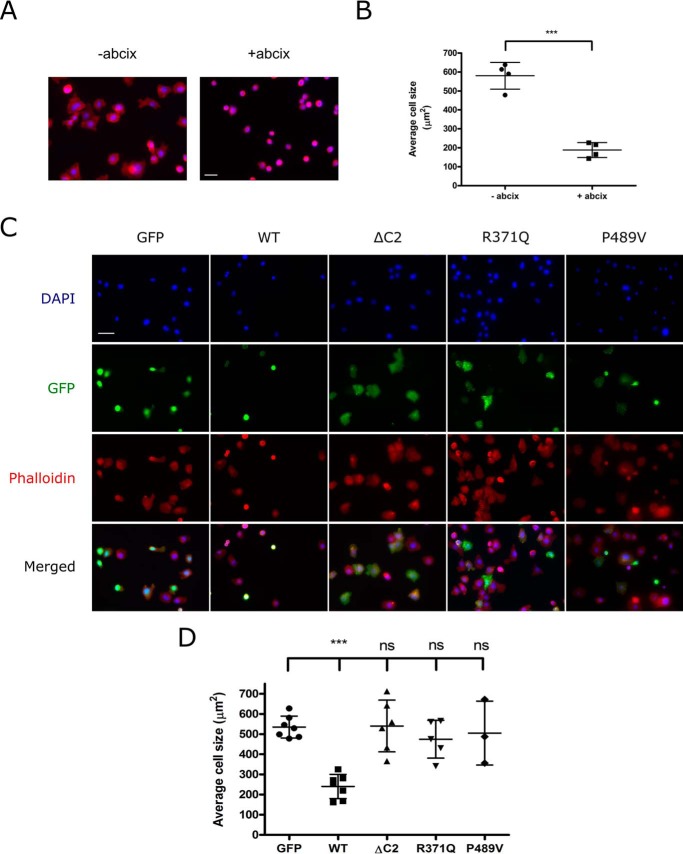
**Integrin α_IIb_β_3_-mediated spreading of CHO cells is inhibited by Rasa3 Rap1GAP activity.** *A* and *B*, CHO cells were allowed to adhere to 100 μg/ml fibrinogen, in the absence or presence of 10 μg/ml abciximab, at 37 °C. Adherent cells were fixed and stained with CruzFluor 594-phalloidin (*red*) and DAPI (*blue*). Images were acquired using a Leica AF6000 wide field microscope at 40× magnification. *A*, representative images of spread CHO cells in the absence (−*abcix*) or presence (+*abcix*) of 10 μg/ml abciximab. *Scale bar*, 32 μm. *B*, cell area was analyzed by measuring the phalloidin staining per cell using ImageJ software. The results are expressed as means ± standard deviation (*n* = 4; ***, *p* ≤ 0.001). *C* and *D*, CHO cells were transfected with GFP alone or GFP-conjugated WT Rasa3, Rasa3-ΔC2, Rasa3 (R371Q), or Rasa3 (P489V) and then allowed to adhere to 100 μg/ml fibrinogen at 37 °C. Adherent cells were fixed and stained with CruzFluor 594-phalloidin (*red*) and DAPI (*blue*). Prior to the spreading assay, CHO cells were transfected with GFP alone or GFP-conjugated WT Rasa3, Rasa3-ΔC2, Rasa3 (R371Q), or Rasa3 (P489V). GFP (*green*) expression indicates transfected cells. The images were acquired using a Leica AF6000 wide field microscope at 40× magnification. *C*, representative images of spread CHO cells transfected with GFP, WT Rasa3, Rasa3-ΔC2, Rasa3 (R371Q), or Rasa3 (P489V). *Scale bar*, 32 μm. *D*, cell area was analyzed by measuring the phalloidin staining per cell using ImageJ software. The results are expressed as means ± standard deviation compared with GFP control (*n* = 3–7; ***, *p* ≤ 0.001).

To evaluate the role of Rasa3 in regulating the activity of Rap1 and Ras, we used an established CHO cell line that stably expresses human integrin α_IIb_β_3_ and tetracycline-inducible thrombin receptor (protease-activated receptor 1 (PAR1)) and talin (27). Endogenous Rasa3 expression was undetectable in these cells (Fig. 1*D*), and GFP-conjugated wild-type Rasa3 predominantly localized to the plasma membrane, as observed for endogenous Rasa3 in platelets (see Fig. 5*A*). Expression of wild-type Rasa3 blocked basal levels of Rap1 activation. In contrast, a mutant form of Rasa3 lacking its N-terminal C2 domains (ΔC2-Rasa3), a Rap1GAP/RasGAP-inactive Rasa3 (Rasa3 (R371Q)), or a Rap1GAP-deficient Rasa3 (Rasa3 (P489V)) had no effect (Fig. 4, *B* and *D*, and Table 1) (19, 28). The PAR1 peptide SFLLRN increased Rap1 activation in CHO cells, which was strongly reduced by wild-type Rasa3 but unaffected by ΔC2-Rasa3, Rasa3 (R371Q), and Rasa3 (P489V) (Fig. 4, *C* and *E*), confirming that wild-type Rasa3 expression causes a reduction in active Rap1-GTP levels specifically through its Rap1GAP activity. Similarly, PAR1 stimulation of CHO cells caused an increase in Ras-GTP, and wild-type Rasa3 overexpression caused a reduction in active Ras levels (Fig. 4, *C* and *E*), although not to the same extent as its effect on active Rap1 (Fig. 4, *B* and *D*). Expression of ΔC2-Rasa3 and Rasa3 (R371Q) had no effect on Ras-GTP levels, whereas the Rap1GAP-deficient P489V mutant had similar effects on reducing Ras activation as wild-type Rasa3 (Fig. 4, *C* and *E*).

**TABLE 1:** **Summary of Rasa3 mutants** The table includes the location of each mutant and a description of the effect the mutation has on Rasa3 localization and activity. The effects on Rasa3 not shown in this study are referenced as appropriate. RasGAP, RasGAP-related domain.

Rasa3	Mouse model	Location	Effect on Rasa3
ΔC2		Deletion of C2 domains	Required to stabilize RasGAP domain (28)
			Loss of GAP activity
R371Q		RasGAP	Loss of GAP activity
P489V		RasGAP	RapGAP-deficient
G125V	*scat*	Between C2 domains	Mislocalization to cytosol (32); loss of GAP activity
H794L	*hlb381*	C terminus	Reduced expression in Rasa3^hlb/hlb^ mice (8); loss of GAP activity

##### Integrin α_IIb_β_3_-dependent Spreading Is Inhibited by the Rap1GAP Activity of Rasa3

To explore the role of Rasa3 in outside-in signaling downstream of integrin α_IIb_β_3_, we performed fibrinogen-spreading experiments of CHO cells transfected with GFP-tagged Rasa3. In platelets, spreading on fibrinogen is a consequence of integrin α_IIb_β_3_-mediated outside-in signaling (29–31). We first confirmed that spreading of these CHO cells on fibrinogen is mediated by integrin α_IIb_β_3_ by blocking spreading using integrin α_IIb_β_3_ antagonist abciximab (Fig. 5, *A* and *B*). Strikingly, expression of GFP-conjugated wild-type Rasa3 blocked spreading of CHO cells on fibrinogen compared with CHO cells expressing GFP alone (Fig. 5, *C* and *D*). In contrast, GFP-tagged forms of ΔC2-Rasa3, Rasa3 (R371Q), or Rasa3 (P489V) had no effect on CHO cell spreading on fibrinogen. These results are not due to changes in receptor expression levels following Rasa3 overexpression because integrin α_IIb_β_3_ subunit and PAR1 levels were unchanged (data not shown). Together, these data indicate that Rasa3-dependent suppression of integrin α_IIb_β_3_-mediated outside-in signaling is through inhibition of Rap1 and not Ras.

##### Rasa3 hlb and scat Mutations Cause a Reduction in GAP Activity and Function

**FIGURE 6.**
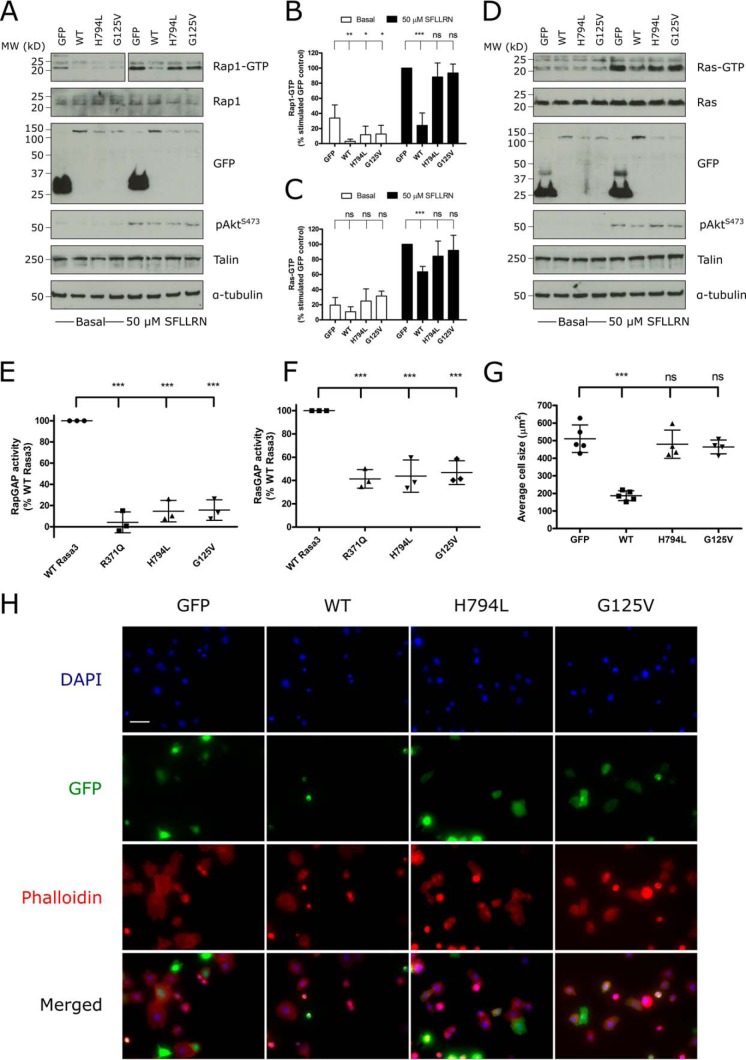
**Rasa3 *hlb* and *scat* forms have deficient RasGAP activity and reduced Rap1GAP activity upon stimulation.** *A–D*, CHO cells were transfected with GFP alone or GFP-conjugated WT Rasa3, Rasa3 (H794L), or Rasa3 (G125V), and Rap1-GTP (*A*) or Ras-GTP (*D*) activation assays were carried out as described for Fig. 4 (*B–E*). *A* and *D*, representative blots from at least four independent experiments. *B* and *C*, quantification of blots, expressed as means ± standard deviation of the percentage of the stimulated GFP control (*B*, *n* = 4–6; *C*, *n* = 4–6) detected. The values are compared with the basal or stimulated GFP control to test for significance (*, *p* ≤ 0.05; **, *p* ≤ 0.01; ***, *p* ≤ 0.001). *E* and *F*, 25 nm recombinant Rasa3 (WT, R371Q, H794L, or G125V) was incubated with [γ-^32^P]GTP-loaded 1 μm Rap1b or H-Ras for 10 min at 25 °C, and GAP activity was measured as described under “Experimental Procedures” (*n* = 3; ***, *p* ≤ 0.001). *G* and *H*, CHO cells were transfected with GFP alone or GFP-conjugated WT Rasa3, Rasa3 (H794L), or Rasa3 (G125V) and then allowed to adhere to 100 μg/ml fibrinogen at 37 °C. Adherent cells were fixed and stained with CruzFluor 594-phalloidin (*red*) and DAPI (*blue*). GFP (*green*) expression indicates transfected cells. Images were acquired using a Leica AF6000 wide field microscope at 40× magnification. *G*, cell area was analyzed as described for Fig. 5*D*. The results are expressed as means ± standard deviation compared with GFP control (*n* = 4–5; ***, *p* ≤ 0.001). *H*, representative images of spread CHO cells transfected with GFP, WT Rasa3, Rasa3 (H794L), or Rasa3 (G125V). *Scale bar*, 32 μm.

Two Rasa3 mutants present in thrombocytopenic mice, Rasa3 (G125V) and Rasa3 (H794L), have recently been described (8, 32). The Rasa3 (G125V) mutant protein is proposed to be cytosolic and thus to have deficient GAP activity (32). The H794L mutation causes a marked reduction in expression of Rasa3 in mice homozygous for this mutation (8). We sought to characterize the effect that these mutations had on Rasa3 GAP activity and integrin α_IIb_β_3_-mediated spreading. When expressed in resting CHO cells, Rasa3 (G125V) and Rasa3 (H794L) inhibited Rap1-GTP levels in a similar manner to wild-type Rasa3 (Fig. 6, *A* and *B*). Under SFLLRN-stimulated conditions, however, Rasa3 (G125V) and Rasa3 (H794L) expression had no effect on Rap1 activation. Expression of Rasa3 (G125V) and Rasa3 (H794L) had no significant effect on Ras activation in resting or stimulated cells (Fig. 6, *C* and *D*). Because the lack of effect of the Rasa3 H794L mutant may potentially be caused by lower total expression levels (Fig. 6, *A* and *D*), we also performed *in vitro* assays using recombinant forms of Rasa3 (G125V) and Rasa3 (H794L), as well as wild-type Rasa3 and GAP-inactive mutant R371Q as a control. As clearly shown, Rasa3 (G125V) and Rasa3 (H794L) are unable to enhance the GTPase function of major platelet Rap1 isoform Rap1b or H-Ras (Fig. 6, *E* and *F*). To test whether the changes in GAP activity caused by the G125V and H794L mutations had an effect on the role of Rasa3 in outside-in signaling, we measured spreading of CHO cells transfected with these mutants. As expected and similar to other Rasa3 mutants with perturbed Rap1GAP activity (Fig. 5, *C* and *D*), the G125V and H794L mutations reduced the ability of Rasa3 to inhibit CHO cell spreading on fibrinogen (Fig. 6, *G* and *H*).

##### Spreading of Rasa3-deficient Platelets Is Insensitive to PI3K Inhibition

**FIGURE 7.**
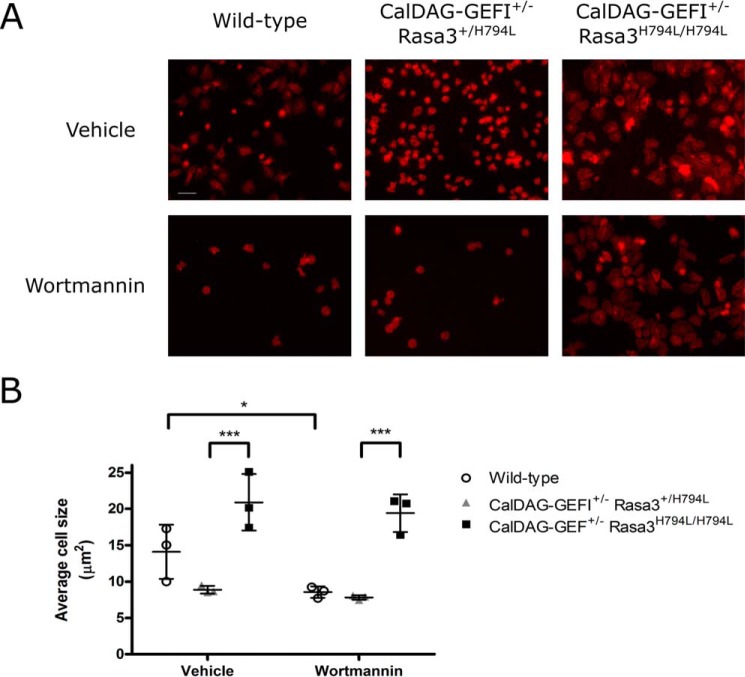
**Spreading of Rasa3^H794L/H794L^ mouse platelets on fibrinogen.** Platelets from CalDAG-GEF1^+/−^ Rasa3^H794L/H794L^ mice were incubated with 100 nm wortmannin or vehicle control for 10 min before being allowed to spread on 100 μg/ml fibrinogen for 60 min at 37 °C. After fixation, platelets were stained with Alexa Fluor 594-phalloidin. *A*, representative images of spread platelets from wild-type, CalDAG-GEF1^+/−^ Rasa3^+/H794L^, and CalDAG-GEF1^+/−^ Rasa3^H794L/H794L^ mice. *Scale bar*, 10 μm. *B*, quantification of platelet spreading by measuring the extent of phalloidin staining per platelet using ImageJ software (*n* = 3).

To further explore the effect of the Rasa3 (H794L) mutation on outside-in signaling, we studied the spreading of murine platelets expressing Rasa3 (H794L). However, Rasa3^H794L/H794L^ mice are severely thrombocytopenic, a phenotype that is rescued by the concomitant deletion of the Rap1 guanine nucleotide exchange factor (GEF), CalDAG-GEFI (8). To circumvent the very low platelet count but not abolish CalDAG-GEFI function, we used platelets from CalDAG-GEFI^+/−^ Rasa3^H794L/H794L^ mice for our spreading experiments (8). After 60 min of exposure to a fibrinogen-coated surface, platelets from CalDAG-GEFI^+/−^Rasa3^H794L/H794L^ mice were extensively spread, as opposed to wild-type or CalDAG-GEFI^+/−^Rasa3^+/H794L^ control platelets that underwent limited spreading (Fig. 7, *A* and *B*). Interestingly, spreading of CalDAG-GEFI^+/−^ Rasa3^H794L/H794L^ mouse platelets was unaffected by treatment with wortmannin, unlike platelets from wild-type mice, demonstrating that PI3K mediates α_IIb_β_3_-mediated spreading through regulation of Rasa3.

## Discussion

We here characterized the Ras/Rap1GAP Rasa3 as a major PI(3,4,5)P_3_ binder and PI3K-regulated protein in human platelets. We have shown for the first time that Rasa3 acts downstream of integrin α_IIb_β_3_ to control cell spreading by inactivating Rap1 and that Rasa3 G125V and H794L mutations found in thrombocytopenic mice have a profound effect on Rasa3 function. Our results support the concept that Rasa3 is closely associated with integrin α_IIb_β_3_ and keeps Rap1 in an inactive form. Integrin-mediated PI3K activity generates PI(3,4,5)P_3_, which leads to an inhibition of Rasa3 GAP activity, allowing Rap1 activation and cell spreading to occur.

The Rap1/RasGAP protein Rasa3 was originally purified and identified from pig platelets in a search for inositol 1,3,4,5-tetrakisphosphate-binding proteins and named GAP1^IP4BP^ (18, 33). Rasa3 is a member of the GAP1 family of proteins that contains a C-terminal pleckstrin homology domain that binds to PI(4,5)P_2_, as well as PI(3,4,5)P_3_, thus targeting Rasa3 to the plasma membrane (25, 34). Research into establishing the role of Rasa3 in platelet function has been hindered by embryonic lethality of the global Rasa3 knock-out mice and severe thrombocytopenia of animal models with impaired Rasa3 expression (8, 20, 35). A spontaneous G125V mutation of Rasa3 was found in *scat* (severe combined anemia and thrombocytopenia) mice, which undergo hematological “crises,” whereby blood cells are depleted and take on a diseased morphology, also causing eventual lethality (32). Furthermore megakaryocytic conditional Rasa3 knock-out mice were also severely thrombocytopenic, and mice with a H794L mutation in Rasa3 showed a drastic reduction in Rasa3 expression and thrombocytopenia (8, 20).

In this study, using an affinity proteomics approach, we identified Rasa3 as one of the major PI(3,4,5)P_3_-binding proteins in human platelets. Furthermore, we found that the majority (∼75%) of Rasa3 is localized at the platelet membrane, which indeed is likely to be mediated through its known interaction with PI(4,5)P_2_ (25, 34). Platelet activation resulted in a net translocation of Rasa3 to the membrane, which was prevented by the pan-PI3K inhibitor wortmannin, the PI3K p110β inhibitor TGX-221, and the P2Y_12_ blocker AR-C66096, demonstrating that PI3K-mediated PI(3,4,5)P_3_ generation results in increased membrane association of Rasa3. This is in agreement with a previous study showing that in HEK cells, PI(3,4,5)P_3_ generation causes the loss of the cytosolic portion of Rasa3 and an increase in Rasa3 plasma membrane association (26). Interestingly, we found a close association between integrin α_IIb_β_3_ and Rasa3 in human platelets, making Rasa3 perfectly positioned to regulate integrin function.

The most likely mechanism by which Rasa3 regulates integrin and platelet function is through its GAP activity toward Rap1 and/or Ras. Of these, Rap1 has a well established function in both inside-out and outside-in regulation of integrins (15, 16, 36), whereas the role of Ras in platelets is currently unknown, but its potential function is an interesting consideration. Previous studies have implicated a negative role of H-Ras in integrin α_IIb_β_3_ activation (37) and, along with this study, have shown that Ras activation occurs in response to thrombin, PKC stimulation, convulxin, U46619, and TPO in platelets (38, 39). However, unlike the regulation of Rasa3, Rap1, and integrin α_IIb_β_3_ in human platelets, we found that Ras activation was not dependent on PI3K, strongly suggesting that PI3K-mediated regulation of Rasa3 is likely to affect Rap1 and not Ras in human platelets. Our results in recombinant CHO cells that constitutively express integrin α_IIb_β_3_ further support a major role of Rasa3 in regulating Rap1 activity downstream of integrin α_IIb_β_3_. Cell spreading on fibrinogen was used as a well established assay for studying integrin α_IIb_β_3_-mediated outside-in signaling independent of inside-out signals and integrin affinity modulation (31, 40). Expression of Rasa3 reduced both Ras and Rap1 activation and blocked integrin-mediated CHO cell spreading on fibrinogen. We confirmed that this effect was mediated through an effect of Rasa3 on Rap1, and not Ras, because Rap1GAP-inactive Rasa3 (P489V) was unable to inhibit CHO cell spreading despite being fully RasGAP-active. Interestingly, we show here that the H794L mutation also leads to impaired Rasa3 GAP activity and loses its ability to block integrin-mediated cell spreading in CHO cells. Furthermore, Rasa3 (G125V), a mutation located between the two C2 domains and present in *scat* mice, was also GAP-inactive. The finding that both Rasa3 (H794L) and (G125V) were both intrinsically Rap1GAP- and RasGAP-inactive was interesting given our previous work showing that Rasa3 C2 domain or C-terminal tail deletion mutants, containing the respective locations of G125 and H794 (Fig. 1*A*), were only Rap1GAP-inactive, retaining full RasGAP activity (28). It therefore seems likely that the H794L and G125V mutations affect Rasa3 protein structure or Ras-binding, significantly diminishing RasGAP activity.

Together, our data demonstrate the important role of Rasa3/Rap1 in integrin-mediated outside-in signaling and cell spreading. Rasa3 is also likely to contribute to inside-out signaling, because platelets from Rasa3^H794L/H794L^ mice had increased Rap1 activity and integrin α_IIb_β_3_ activation (8). Crossing Rasa3^H794L/H794L^ mice with mice deficient in the RapGEF CalDAG-GEFI reversed increased platelet integrin activation and partially normalized platelet count and life span (8), demonstrating that Rasa3 regulation of Rap1 underlies the phenotype. To confirm the role of Rasa3 in outside-in signaling in platelets, we utilized the Rasa3^H794L/H794L^ mouse model, with a slight variation in that they were also heterozygous for CalDAG-GEFI to ensure sufficient platelet numbers (8). CalDAG-GEFI^+/−^Rasa3^H794L/H794L^ platelets exhibited increased spreading on fibrinogen, demonstrating that Rasa3 prevents spreading downstream of integrin engagement.

**FIGURE 8.**
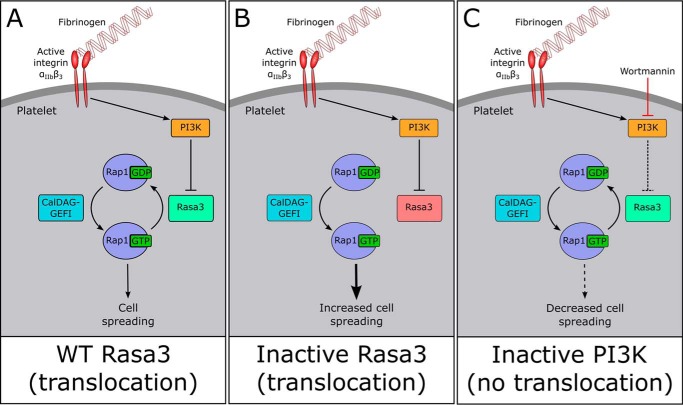
**Role of Rasa3 and PI3K in platelet integrin α_IIb_β_3_ outside-in signaling.** *A*, fibrinogen binding to activated integrin α_IIb_β_3_ initiates outside-in signaling, including the activation of PI3K. PI3K reduces Rasa3 Rap1GAP activity, thus allowing CalDAG-GEFI-mediated Rap1 activation to occur uninhibited. Activated GTP-bound Rap1 then promotes further signaling processes that lead to cell spreading. *B*, Rap1GAP-inactive forms of Rasa3 (shown in “*red*”), such as Rasa3 (H794L), are unable to mediate Rap1 inactivation, leading to enhanced cell spreading. *C*, inhibition of PI3K by wortmannin releases the Rap1GAP activity of Rasa3, thus enabling the conversion of Rap1 into its inactive GDP-bound state. As a result, cell spreading is severely reduced. Note that the role of PI3K in platelet spreading is upstream of Rasa3, and therefore cells containing intrinsically Rap1GAP-inactive forms of Rasa3 are insensitive to PI3K inhibition.

This result corroborates with previous work showing the involvement of Rap1 in integrin-mediated spreading and the finding that platelets from patients with a mutation in CalDAG-GEFI have deficient spreading (17, 36, 41, 42). It is well established that integrin α_IIb_β_3_ outside-in signaling and subsequent cell spreading is dependent on PI3K (this study and Ref. 4), and we hypothesize that one of the major mechanisms by which PI3K regulates outside-in signaling is by inhibiting Rasa3 Rap1GAP activity (Fig. 8). Indeed, the importance of PI3K in the regulation of Rasa3 downstream of integrin α_IIb_β_3_ was clearly demonstrated by the insensitivity of integrin α_IIb_β_3_-mediated spreading of CalDAG-GEFI^+/−^Rasa3^H794L/H794L^ platelets to a PI3K inhibitor.

Taken together, our results provide new insight into the mechanism by which PI3K regulates platelet function, in particular by controlling Rasa3 downstream of integrin α_IIb_β_3_. We propose that PI3K regulates Rap1 activation downstream of integrin α_IIb_β_3_ by inhibition of Rasa3 Rap1GAP activity, leading to sustained Rap1 activation and cell spreading.

## Experimental Procedures

### 

#### 

##### Materials

Goat anti-Rasa3 (sc-34468), rabbit anti-Rap1 (sc-65), goat anti-Btk (Bruton's tyrosine kinase) (sc-1107), mouse anti-integrin α_IIb_β_3_ (sc-21783), and goat anti-talin (sc-7534) antibodies were obtained from Santa Cruz Biotechnology (Insight Biotechnology, Wembley, UK). Rabbit anti-FcRγ (06–727) and mouse anti-Ras (05–516) (recognizing p21 H-, K-, and N-Ras) antibodies were from Millipore (Billerica, MA). Rabbit anti-pS^473^ Akt (4060), rabbit anti-Akt (9272), rabbit anti-ERK (9102), and rabbit anti-GFP (2555) antibodies were sourced from Cell Signaling Technologies (New England Biolabs, Hitchin, UK), and the rat anti-GPIX (M051-0) antibody was from Emfret Analytics (Wuerzburg, Germany). PE-conjugated mouse anti-CD61 (555754) antibody was from BD Biosciences (Oxford, UK), and PE-conjugated mouse anti-PAR1 (IM2584) antibody and DRAQ7 were from Beckman Coulter (High Wycombe, UK). Secondary peroxidase-conjugated antibodies were from Jackson Immunoresearch (Stratech, Newmarket, UK). Control and PI(3,4,5)P_3_-coated beads were from Tebu-bio (Peterborough, UK). Alexa Fluor 350/488/568-conjugated secondary antibodies, PE-conjugated mouse anti-CD41 (MHCD4104) and mouse IgG_1_ (MG104) antibodies Lipofectamine 2000 and NuPAGE LDS sample buffer were obtained from Life Technologies (Carlsbad, CA). Enhanced chemiluminescent materials, GSH-Sepharose beads, and PD-10 desalting columns were sourced from GE Healthcare. PAR1-activating peptide (SFLLRN) was from Bachem (Weil am Rhein, Germany), and abciximab was from Eli Lily (Basingstoke, UK). cOmplete Mini protease inhibitor tablets were from Roche Applied Science, and microcystin-LR was from Axxora (Nottingham, UK). BL21(DE3)pLysS competent cells were obtained from Promega (Southampton, UK). AR-C 66096 and wortmannin were from Tocris (Avonmouth, UK). TGX-221 was from Selleckchem (Houston, TX). [γ-^32^P]GTP was from PerkinElmer Life Sciences. Fibrinogen used in the mouse spreading experiments was sourced from Enzyme Research Laboratories (South Bend, IN). All other reagents were obtained from Sigma-Aldrich unless stated otherwise.

##### Construction of Rasa3 Mutants

Rasa3-pEGFP-C1 and ΔC2Rasa3-pEGFP-C1 have been described previously (25). Site-directed mutagenesis of Rasa3 in pEGFP-C1 was carried out as described by the manufacturer (QuikChange II; Agilent Technologies) using the following primers: 5′-cccaggatcccaacaccatcttccaaggaaactc-3′ (R371Q), 5′-ggttctttgcggtcgcgattctctccccc-3′ (P489V), 5′-gactcggaagtgcaggtcaaagtgcacctggag-3′ (G125V), and 5′-GGGGCTTTGGAGCAGGAGCTCGCCCAGTATAAGAGGGACAA-3′ (H794L). All mutated codons are underlined and were confirmed by DNA sequencing.

##### Preparation of GST-tagged RafGDS-RBD and RalGDS-RBD

Plasmids encoding GST-RafGDS-RBD and GST-RalGDS-RBD were heat shock transfected into BL21(DE3)pLysS competent cells and grown on agar plates containing LB-Amp (Luria-Bertani + 50 μg/ml ampicillin). Single colonies of transformed bacteria were grown for 24 h at 37 °C before being diluted in 1 liter of LB-Amp. Protein was induced with the addition of 1 mm isopropyl β-d-thiogalactopyranoside for 3 h, and bacteria were pelleted (6000 × *g*, 20 min, 4 °C) and stored at −80 °C overnight. Bacteria resuspended in RBD buffer (PBS, 5 mm MgCl_2_, 1% Triton X-100, 1 mm PMSF, 5 mm DTT, protease inhibitors) were sonicated and tumbled with GSH-Sepharose beads for 30 min. The beads were pelleted (500 × *g*, 5 min, 4 °C), washed with storage buffer (RBD buffer + 5% glycerol), resuspended in elution buffer (RBD buffer + 15 mm reduced l-glutathione), and loaded onto PD-10 desalting columns. Eluates containing the GST-tagged proteins were collected by washing the columns with storage buffer.

##### Isolation of Primary Cells and Cell Culture

Human platelets (13), mouse platelets (8, 43) and megakaryocytes (44) were prepared as described previously. Peripheral blood mononuclear cells were isolated with Histopaque (Sigma-Aldrich) according to the manufacturer's instructions. CHO-K1 cells expressing αIIbβ3 and inducible PAR1/talin (gift from S. Shattil) were cultured as described (27). The cells were transfected with Lipofectamine (Life Technologies) following the manufacturer's protocol, followed by doxycycline treatment to induce PAR1/talin expression for 24 h.

##### Capture of PI(3,4,5)P_3_-binding Proteins from Human Platelet Lysates

Resting platelets were pelleted at 520 × *g* for 10 min and lysed in ice-cold lysis buffer (20 mm HEPES, pH 7.4, at 4 °C, 120 mm NaCl, 0.5% Nonidet P-40, 5 mm EGTA, 5 mm EDTA, 5 mm β-glycerophosphate, 10 mm NaF, 1 mm Na_3_VO_4_, and protease inhibitors). Following vortexing and tumbling for 20 min at 4 °C, the lysates were centrifuged at 16,000 × *g* for 10 min at 4 °C. The resulting supernatants were incubated in the presence or absence of 40 μm PI(3,4,5)P_3_ for 20 min at 4 °C under gentle rotation, before addition to 30 μl of pre-equilibrated control or PI(3,4,5)P_3_ beads for 90 min at 4 °C under gentle rotation. The beads were washed three times with ice-cold lysis buffer. The proteins were processed for Western blotting or for mass spectrometry.

##### Mass Spectrometry

Proteomics was performed as previously described (45), with a few modifications. A single gel slice for each pulldown was subjected to in-gel tryptic digestion using a ProGest automated digestion unit (Digilab UK). The resulting peptides were fractionated using a Dionex Ultimate 3000 nanoHPLC system in line with an LTQ-Orbitrap Velos mass spectrometer controlled by Xcalibur 2.1 software (Thermo Scientific) operated in data-dependent acquisition mode. The raw data files were processed and quantified using Proteome Discoverer software v1.2 (Thermo Scientific) and searched against the UniProt Human database (122604 sequences) using the SEQUEST (Ver. 28 Rev. 13) algorithm. The reverse database search option was enabled, and all peptide data were filtered to satisfy a false discovery rate of 5%.

##### Protein Extraction and Immunoblotting

Washed platelets were incubated with 100 nm wortmannin, 1 μm AR-C 66096 or vehicle for 10 min and stimulated with 0.2 unit/ml thrombin for the indicated time. Alternatively, CHO cells were stimulated with 50 μm SFLLRN for 5 min. The samples were lysed in ice-cold 2× radioimmune precipitation assay buffer (50 mm HEPES, pH 7.4, 400 mm NaCl, 2 mm EDTA, 2% (v/v) Nonidet P-40, 1% (w/v) sodium deoxycholate, 0.2% (w/v) SDS, 40 mm sodium β-glycerophosphate, 20 mm sodium pyrophosphate, 2 mm benzamidine, protease inhibitors, 10 mm Na_3_VO_4_, and 2 μm microcystin-LR) for whole cell lysates or ice-cold 2× Rap1 lysis buffer (50 mm HEPES, pH 7.4, 400 mm NaCl, 5 mm MgCl_2_, 2% (v/v) Nonidet P-40, 20% glycerol, protease inhibitors, 10 mm Na_3_VO_4_, and 2 μm microcystin-LR) for pulldown samples. Lysates were incubated on ice for 30 min, and proteins were extracted in the supernatant of centrifuged lysates (4 °C, 16,200 × *g*, 10 min). A Bradford assay was performed to determine the approximate protein concentration of whole cell lysates. For Western blotting, lysates were mixed with 4× sample buffer (NuPAGE LDS sample buffer + 50 mm DTT) and processed for SDS-PAGE and immunoblotting as previously described (46).

##### Rap1 and Ras Activation Assays

Purified GST-RafGDS-RBD and GST-RalGDS-RBD proteins were bound to GSH-Sepharose beads overnight at 4 °C. Pulldown samples in Rap1 buffer were incubated on ice for 20 min to complete extraction, and proteins were extracted in the supernatant of centrifuged lysates (16,200 × *g*, 10 min, 4 °C). Platelet lysates were tumbled with 20 μg of immobilized GST-RafGDS-RBD or GST-RalGDS-RBD for 1 h at 4 °C to pull down Ras-GTP or Rap1-GTP, respectively. The beads were repeatedly pelleted (4 °C, 16,200 × *g*, 20 s) and washed with Rap1 buffer before elution in NuPAGE LDS sample buffer.

##### Platelet Fractionation

Stimulated platelets were diluted in an equal volume of sonication buffer (50 mm Tris, pH 7.4, 250 mm sucrose, protease inhibitors, 10 mm Na_3_VO_4_, and 2 μm microcystin-LR) and sonicated (five times for 20 s). The lysate was centrifuged (1500 × *g*, 10 min, 4 °C) to remove intact platelets, and the membrane fraction was pelleted (100,000 × *g*, 2 h, 4 °C). The supernatant (cytosolic fraction) was removed, and the pellet was resuspended in radioimmune precipitation assay buffer.

##### Immunofluorescence

Stimulated and untreated platelets were fixed in 4% formaldehyde and spun onto glass coverslips (180 × *g*, 5 min). Adhered platelets were washed with PBS and permeabilized with 0.1% Triton X-100. 1% fatty-acid free BSA was added for 1 h at room temperature before overnight incubation with primary antibodies (1:500) at 4 °C. Excess antibody was washed off, and the samples were incubated with Alexa Fluor 350/488/468-conjugated anti-rabbit/goat/mouse antibodies for 1 h at room temperature. Coverslips were mounted onto slides and imaged with a 100× oil objective lens (numerical aperture, 1.4) using a spinning disk confocal module (PerkinElmer UltraVIEW ERS 6FE confocal microscope) equipped with a C9100–50 EM-CCD camera (Hamamatsu). Analysis was performed using Volocity software (PerkinElmer) on at least 20 cells, with submembrane defined as 0.5 μm from the outermost point of the cell.

##### CHO Cell Spreading Assay

Coverslips were coated with 100 μg/ml fibrinogen overnight at 4 °C followed by the addition of 2% (w/v) fatty-acid free BSA for 2 h at 37 °C. 1 × 10^6^ transfected CHO cells were allowed to adhere to fibrinogen for 30 min at 37 °C. Some cells were incubated with 10 μg/ml abciximab prior to adhesion. Non-adherent cells were washed away, and cells were fixed in 4% formaldehyde for 10 min, permeabilized with 0.1% Triton X-100, and stained with CruzFluor 594-phalloidin and DAPI. Images were acquired using a Leica AF6000 wide field microscope equipped with a dry 40× objective lens (numerical aperture, 0.6) and a DFC365FX monochrome CCD camera (Leica). Cell area was analyzed by measuring the phalloidin staining per cell using ImageJ software.

##### Mouse Platelet Spreading Assay

Glass-bottomed plates were coated with 100 μg/ml fibrinogen for 1 h followed by the addition of 3% (w/v) BSA for 30 min. Washed mouse platelets at 7.5 × 10^7^/ml were allowed to adhere to fibrinogen for the indicated time at 37 °C in the presence of 2 mm Ca^2+^. Some cells were incubated with 100 nm wortmannin or vehicle control prior to adhesion. The cells were fixed in an equal volume of 4% formaldehyde for 10 min, permeabilized with 0.1% Triton X-100, and stained with Alexa Fluor 647-GPIX antibody and Alexa Fluor 594-phalloidin to visualize platelets and F-actin, respectively. Image acquisition and cell area quantification were performed as above.

##### GAP Activity Assays

Assays to determine Rasa3 (H794L) and (G125V) GAP activity were performed as previously described under first order kinetics and using 25 μm recombinant Rasa3 per assay (18). Each experiment was performed in triplicate.

##### Imaging Rasa3 Localization in CHO Cells

CHO cells were seeded onto poly-l-lysine-coated glass-bottomed dishes (1 × 10^5^ cells/dish) overnight under normal growing conditions, before the cells were transfected with GFP or GFP-conjugated wild-type Rasa3. 16 h after transfection, the adhered cells were washed twice in imaging medium (phenol red-free DMEM, 25 mm HEPES, 10% FCS) and incubated at 37 °C without CO_2_. The cells were imaged with a 63× glycerol objective lens (numerical aperture, 1.3) using the previously described spinning disk confocal module and camera.

##### Flow Cytometry

CHO cells were harvested and resuspended in FACS Tyrode's (12.1 mm NaHCO_3_, 10 mm HEPES, 137 mm NaCl, 2.6 mm KCl, 5.6 mm glucose, 1 mg/ml BSA) containing 1 mm CaCl_2_ and 1 mm MgCl_2_. CHO cells were then incubated with 200 ng of PE-conjugated CD41, CD61, PAR1, or isotype control for 30 min on ice. Samples were analyzed on a FACSCanto II flow cytometer and gated for living (DRAQ7^−^) and transfected (GFP^+^) cells.

##### Statistics

The data were analyzed using GraphPad Prism software. All error bars show the means ± standard deviation. Statistical analysis is presented as paired Student's *t* test or analysis of variance (one-way or two-way) followed by Dunnett's post test (*, *p* ≤ 0.05; **, *p* ≤ 0.01; ***, *p* ≤ 0.001).

## Author Contributions

A. M. B. designed and performed research, collected and analyzed data, and wrote the paper. T. N. D. performed research, analyzed data, contributed to discussion, and edited the paper. E. O. A. performed research and analyzed data. K. J. H. provided proteomics services. D. S. P. and R. P. contributed reagents and supported spreading experiments. A. W. P. contributed to discussion. P. J. C. and W. B. provided reagents and contributed to discussion. S. F. M. designed and cosupervised research, performed research, contributed to discussion, and edited the paper. I. H. designed and supervised research, contributed to discussion, and wrote the paper. All authors reviewed the results and approved the final version of the manuscript.

## Footnotes

2 The abbreviations used are:

PI(3,4,5)P_3_: phosphatidylinositol 3,4,5-trisphosphate
Btk: Bruton's tyrosine kinase
GAP: GTPase-activating protein
GEF: guanine nucleotide exchange factor
PAR1: protease-activated receptor 1
PI(4,5)P_2_: phosphatidylinositol 4,5-bisphosphate
PE: phosphatidylethanolamine.
